# Effects of Chromium Carbide Coatings on Microstructure and Thermal Conductivity of Mg/Diamond Composites Prepared by Squeeze Casting

**DOI:** 10.3390/ma15041284

**Published:** 2022-02-09

**Authors:** Jianwei Li, Ren Peng, Jinming Ru, Jianhua Wu, Kaixiang Zhou, Yongxin Yan, Xiaojing Xu, Yuhua Zhou

**Affiliations:** 1 Institute of Advanced Manufacturing and Modern Equipment Technology, Jiangsu University, Zhenjiang 212013, China; jwli4764@ujs.edu.cn (J.L.); 2221903048@stmail.ujs.edu.cn (R.P.); 2221803079@stmail.ujs.edu.cn (K.Z.); 2222003135@stmail.ujs.edu.cn (Y.Y.); xjxu67@126.com (X.X.); 2 Jones PLC Tech Yixing, Tengfei Road, Yixing 21420, China; hxsj116@163.com; 3 Advanced Materials Institute, Qilu University of Technology (Shandong Academy of Sciences), Jinan 250014, China; jianhw@sdas.org

**Keywords:** Mg/diamond composites, thermophysical properties, interface, squeeze casting

## Abstract

Magnesium matrix composites are considered a desired solution for lightweight applications. As an attractive thermal management material, diamond particle-reinforced Mg matrix (Mg/diamond) composites generally exhibit thermal conductivities lower than expected. To exploit the potential of heat conduction, a combination of Cr coating on diamond particles and squeeze casting was used to prepare Mg/diamond (Cr) composites. The thickness of the Cr coating under different coating processes (950 °C/30 min, 950 °C/60 min, 950 °C/90 min, 1000 °C/30 min, and 1050 °C/30 min) was measured by FIB-SEM to be 1.09–2.95 μm. The thermal conductivity (TC) of the Mg/diamond composites firstly increased and then decreased, while the coefficient of thermal expansion (CTE) of Mg/diamond (Cr) composite firstly decreased and then increased with the increase in Cr coating thickness. The composite exhibited the maximum TC of 202.42 W/(m·K) with a 1.20 μm Cr coating layer, while a minimum CTE of 5.82 × 10^−6^/K was recorded with a coating thickness of 2.50 μm. The results clearly manifest the effect of Cr layer thickness on the TC and CTE of Mg/diamond composites.

## 1. Introduction

The rapid development of the electronic field urgently requires the development of thermal management materials with high performance [1,2]. Carbon materials, including carbon fibers, graphite, diamond, carbon foams, carbon nanotubes, graphene, and reinforced metal matrix composites (MMCs) with high TC and low CTE, are the possible solution for electronic packaging applications [3,4]. Among them, diamond is an ideal reinforcement material due to its excellent intrinsic thermal conductivity (1200–2000 W/(m·K)) and low thermal expansion coefficient (2.3 × 10^−6^/K) [5,6].

In the context of diamond-reinforced MMCs with high TC, magnesium and its alloys have long been neglected because of their intrinsic thermal conductivity being lower than that of Al and Cu. Nevertheless, it still needs to be pointed out that the density of magnesium is much lower than that of aluminum and copper. Therefore, the development of diamond-reinforced magnesium matrix composites with high TC and low CTE can further broaden the applications of magnesium alloys [7,8].

Sound interfacial bonding is the basic prerequisite for achieving a metal/diamond composite with high TC. In order to improve the thermal conductivity, diamond surface coating [9,10,11] and metal matrix alloying [12,13,14] have been applied to enhance the interfacial bonding between the diamond and the metal matrix. The surface coating method excels in improving composite performance; Kumar et al. developed a series of zinc-based coatings such as Zn–WO_3_ [15] and Zn–Ni–WC composite nanocoatings [16], which exhibit excellent corrosion resistance and high hardness, greatly expanding the application of steel in boat structures, manufacturing, nuclear power plants, etc. Studies have shown that the addition of alloying elements can drastically reduce the TC of the magnesium matrix [17]. However, it is a feasible method to coat Ti, Mo, W, Zr, Al, Cr, and other carbide-forming elements onto the diamond surface to improve the wettability between the Mg matrix and the diamond [18]. Moreover, it has been reported that Cr coatings have good mechanical stability [19] and thermal conductivity [20].

To the best of our knowledge, there are few reports on the TC of Mg/diamond composites. Stevenson [21] prepared a Mg–5.5Zn–0.5Zr/diamond composite and found that the TC of the composite was not improved by the addition of alloying elements in the matrix. Pickard [22] claimed in his patent that, when the diamond volume fraction is 54%, the TC of the composite can be increased from 120–250 W/(m·K) to 520–550 W/(m·K) by coating SiC on the diamond surface. Molina et al. [7] coated Ti on the diamond surface and prepared Mg/diamond composites by gas pressure infiltration, thereby greatly improving the thermal conductivity of the composite. Zhu et al. [11] used a molten salt method to successfully prepare surface-gradient-modified diamond particles. The value of the thermal conductivity of the surface-modified Mg/diamond composites with a diamond volume percentage of 35% reached 286 W/(m·K). Ma et al. [23] studied the thermal conductivity and mechanical properties of ND-reinforced ZK60 matrix composites; the thermal conductivity of the composite material exceeded 129 W/(m·K) with an ND content of 0.05%. In these cases, the TC values of the Mg/diamond composites were lower than the predicted values. It is, hence, necessary to understand the influence of interfacial carbide evolution on the thermal conductivity.

Numerous efforts have been devoted to improving the thermal conductivity of metal matrix composites; however, there is a paucity of research literature on magnesium-based composites. The commonly used coating methods for diamond surfaces include the molten salt method, vacuum micro-evaporation, chemical vapor deposition, magnetron sputtering, and sol–gel [24,25]. The molten salt method is one of the most commonly used coating methods due to its low cost. However, the effect of the molten salt method on the structure of chromium coating and the TC of Mg/diamond composites has not been studied yet. Therefore, solving this problem will accelerate the application of high-TC electronic packaging materials in the semiconductor, integrated circuit, and aerospace industries.

In the present work, we coated Cr on diamond particles to improve the interface bonding and prepared the composites using the squeeze casting infiltration process. The thickness of the coating in the range 1.09–2.95 μm was measured by FIB-SEM to explore the effect of coatings with different thicknesses on the thermal properties of the Mg/diamond composites.

## 2. Materials and Methods

Commercially available bulk Mg (purity: 99.95 wt.%, Shanxi Yinguang Huasheng Magnesium Co., Yuncheng, China) was used as a metal matrix, and HHD90-type synthetic single-crystalline diamond powders (particle size: 212–250 μm, Henan Huanghe Whirlwind Co., Changge, China) were used to reinforce the composite. NaCl and KCl were used as analyitical reagents (Sinopharm Chemical Reagent Co., Ltd., Zhenjiang, China). NaCl and KCl with a molar ratio of 1:1 were mixed and used to cover the diamond particles for the salt bath. Two different sizes of alumina crucibles were nested and used, and the gap was filled with mixed salt.

### 2.1. Preparation of Cr Coating on Diamond Powder

The Cr coating was deposited onto the diamond particles using the molten salt method. When the temperature is higher than 850 °C, the diamond particles react with Cr powder to form carbides [26]. Thus, the coating temperature and coating time were 950–1050 °C and 30–90 min. The coating process can be described by the following steps: (i) the diamond particles were washed with a diluted acid to remove impurities; (ii) the salt and diamond/Cr powder mixture was placed in an alumina crucible, heating to different temperatures (950 °C, 1000 °C, and 1050 °C) for 30–90 min in a tube furnace, before furnace-cooling to room temperature; (iii) after cooling, the mixture was separated by an ultrasonic wave with boiling distilled water and alcohol, and then the coated diamond particles were dried under vacuum at 100 °C for 30 min.

### 2.2. Preparation of Mg/Diamond (Cr) Composites

The Mg/diamond composites were prepared using the squeeze casting infiltration process. The main process included two steps. Firstly, the Cr-coated diamond particles were densely packed in a graphite mold, and the pure Mg bulks were placed on top of the diamond preform in a quartz crucible. Secondly, considering that pure magnesium is easily oxidized and magnesium liquid can splash easily, the crucible was placed in a resistance furnace, heated to 800 °C, and kept for 10 min under a SF_6_ + CO_2_ protective gas mixture (volume ratio of SF_6_/CO_2_ = 1:99). Then, a uniaxial pressure (10 MPa) was held for 60 s at 800 °C until the infiltration process was accomplished. Finally, the sample was obtained after the graphite mold was cooled to room temperature. The specific raw materials and experimental parameters are shown in Table 1.

### 2.3. Characterization

The microstructures of the uncoated diamond particles, the coated diamond particles, and the diamond extracted from the composite material were observed with a field-emission scanning electron microscope (FE-SEM, JSM-7001F, JEOL Ltd., Tokyo, Japan). The phase compositions of the diamond (Cr) particles and the Mg/diamond (Cr) composites were characterized by X-ray diffraction with Cu-Kα radiation (XRD, D8-ADVANCE, Burker Co., Karlsruhe, Germany). The cross-section of a layer was prepared using a focused ion beam system (FIB-SEM, ZEISS Crossbeam 350, Zeiss Co., Oberkochen, Germany), and the coating thickness was also obtained.

The thermal conductivity of the composites was calculated using the equation $K_{c}=\alpha \cdot \rho_{c}\cdot c$, where $K_{c},\;\alpha ,\;\rho_{c},\;\mathrm{and}\;c$ represent thermal conductivity, thermal diffusivity, sample density, and heat capacity, respectively. The thermal diffusivity was measured using a laser flash apparatus (LFA 457, NETZSCH Group, Selb, Germany) at room temperature with disc-shaped samples of Φ12.7 mm × 2.5 mm. The density of the composite material was measured using the Archimedes drainage method, with absolute ethanol as the liquid medium, an analytical balance with an accuracy of 0.1 mg, and the attached density measuring component. The specific heat capacity of the composite material can be calculated theoretically from the mass fraction of each component using the equation $C=C_{diamond}\cdot W_{diamond}+C_{Mg}\cdot W_{Mg}$, where $C_{diamond}$ and $C_{Mg}$ represent the specific heat capacity of diamond and Mg, and $W_{diamond}$ and $W_{Mg}$ represent the mass fraction of diamond and Mg, respectively. The above variables were averaged from three measurements to calculate the thermal conductivity of the Mg/diamond composites. The thermal expansion coefficient of the composite material was measured in the range 300–573 K with a heating rate of 10 K·min^−1^ in an argon atmosphere using a cuboid sample of 15 mm × 3 mm × 3 mm machined by laser cutting.

## 3. Results

**Figure 1:**
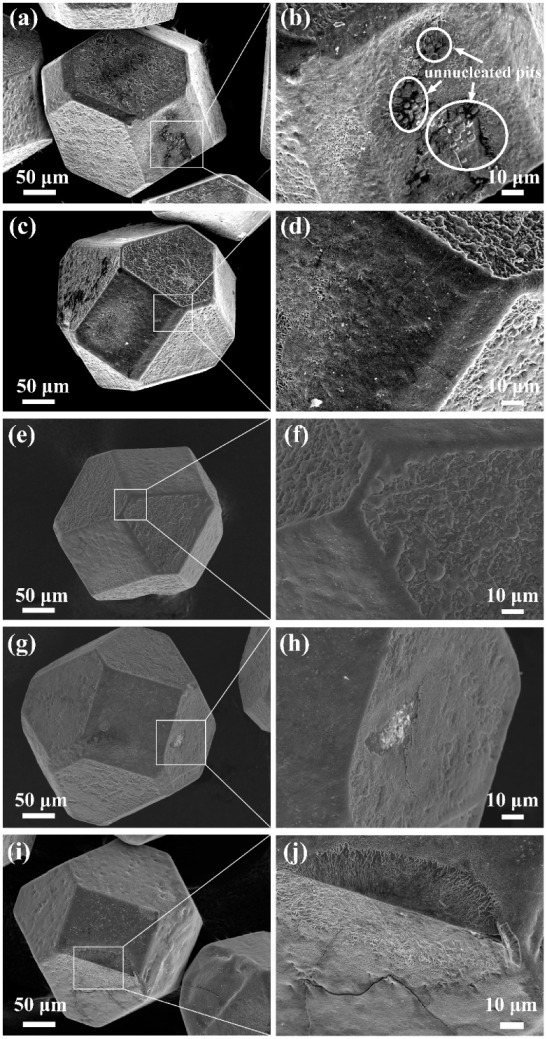
Cr coating diamond surface morphology under salt bath process with different parameters: (**a**,**b**) 950 °C/30 min, (**c**,**d**) 950 °C/60 min, (**e**,**f**) 950 °C/90 min, (**g**,**h**) 1000 °C/30 min, and (**i**,**j**) 1050 °C/30 min.

Figure 1 shows SEM images of the Cr-coated diamond particles. When the coating temperature reached 950 °C, the Cr coating successfully adhered to the diamond surface, and the thickness increased with holding time. However, most of the diamond surface coating structure was incomplete at 950 °C, and almost all coatings were cracked, as shown in Figure 1a. With an increase in the holding time (90 min), the irregular island-like structure of the diamond (100) crystal plane became smoother and denser, and the number of cracks on the diamond surface decreased significantly, as shown in Figure 1e. This shows that, under these process conditions, an appropriate increase in holding time increases the thickness of the coating and optimizes the bond strength between the coating and the diamond surface, thus making the coating more complete.

We also investigated the effect of temperature on the formation of the coating. As the coating temperature was increased, the diamond surface gradually formed a complete coating. At 950 °C, the diamond (111) surface was rough but complete, while the (100) surface had unnucleated pits, and the coating was broken. Moreover, the reaction between diamond and Cr was anisotropic. The (100) crystal plane of diamond had higher activity and reacted more easily with metallic Cr than the (111) face. Above 1000 °C, the edges and corners between the diamond crystal planes become rounded, and the surface roughness decreased; in particular, the coating on the (100) crystal plane became complete. This shows that the increase in temperature sped up the diffusion and deposition of Cr to the diamond surface in the molten salt, such that the thickness of the coating was increased continuously. As the coating temperature further increased to 1050 °C, the originally uniform and dense coating thickened while cracks also developed, and some powder-like substance appeared on the (100) crystal plane, as shown in Figure 1i. It can be seen that diamond particles prepared at too high a salt bath temperature caused coating cracking due to the excessive temperature difference in the subsequent extraction process. At the same time, when the thickness of the coating was increased to a certain extent, it hindered the continuous reaction of Cr and diamond. A certain amount of Cr powder remained on the surface of the coating. From the above analysis, it can be found that the formation of the diamond surface coating is controlled by the diffusion mechanism, and the temperature has an important influence on the microscopic morphology of the coating.

This shows that an excessive temperature and hold time would lead to cracks in the coating in the subsequent extraction process. When the thickness of the coating reaches a certain level, it hinders further reaction between the chromium and the diamond. It can be concluded that temperature and hold time have an important influence on the formation and microscopic morphology of the coatings.

In the coating process, the diamond powder and Cr come into contact and react. In the temperature range 298–1800 K, the Gibbs energies of the reaction between diamond and Cr powder were as shown in Table 2. When the coating temperature was 950 °C, 1000 °C, and 1050 °C, the Gibbs free energy of all reactions was negative, indicating that the three carbides of Cr_3_C_2_, Cr_7_C_3_, and Cr_23_C_6_ could form spontaneously.

**Figure 2:**
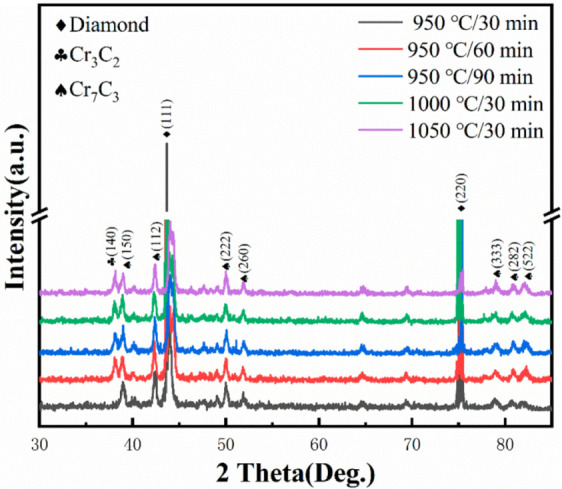
XRD patterns of diamond particles coated at 950–1050 °C.

In order to determine the phase composition of the diamond, we conducted XRD analysis as shown in Figure 2. The diffraction peaks of Cr_3_C_2_ and Cr_7_C_3_ appeared at the coating temperature of 950 °C. However, that of Cr_23_C_6_ was not found, and it can be seen that the change in holding time only changed the intensity of each diffraction peak. With increasing coating temperature, C atoms detached from the coating and participated in the reaction with Cr atoms to form carbides with higher C content. The intensity of the two diffraction peaks did not change much at 1000 °C and 1050 °C, but the amount of Cr_7_C_3_ was significantly greater than that of Cr_3_C_2_. Combined with the Gibbs free energy analysis, the results show that Cr_7_C_3_ was easier to generate than Cr_3_C_2_ at the same temperature. This is also consistent with the results of thermodynamic analysis. During the reaction of diamond and Cr, Cr_23_C_6_ was the first product formed, which was then gradually transformed into Cr_7_C_3_ and Cr_3_C_2_ with the increase in temperature, but the content of Cr_7_C_3_ was significantly higher than that of Cr_3_C_2_.

The film thickness of the Cr-coated diamond particles prepared under different processes was characterized and measured to more accurately quantify the influence of the coating process on the thickness of the coating. In previous studies, researchers mainly used SEM observation and the spherical weight gain method to estimate the thickness of the film [7,27]. However, it is difficult to directly observe the thickness of the film under a scanning electron microscope due to the typical hexahedral–octahedral structure of diamond, leading to the theoretical calculation method having an error. Therefore, in this paper, a focused ion beam (FIB) and scanning electron microscope (SEM) were coupled into an FIB-SEM dual-beam system. The high-current ion beam was used to strip the atoms on the diamond surface to achieve the interception of the microscopic cross-section of the coating structure, and then the cross-sectional layer shape was observed by scanning electron microscope, while the cross-sectional layer was analyzed by EDS line scanning. According to the change in element content, the thickness of the coating was accurately determined.

**Figure 3:**
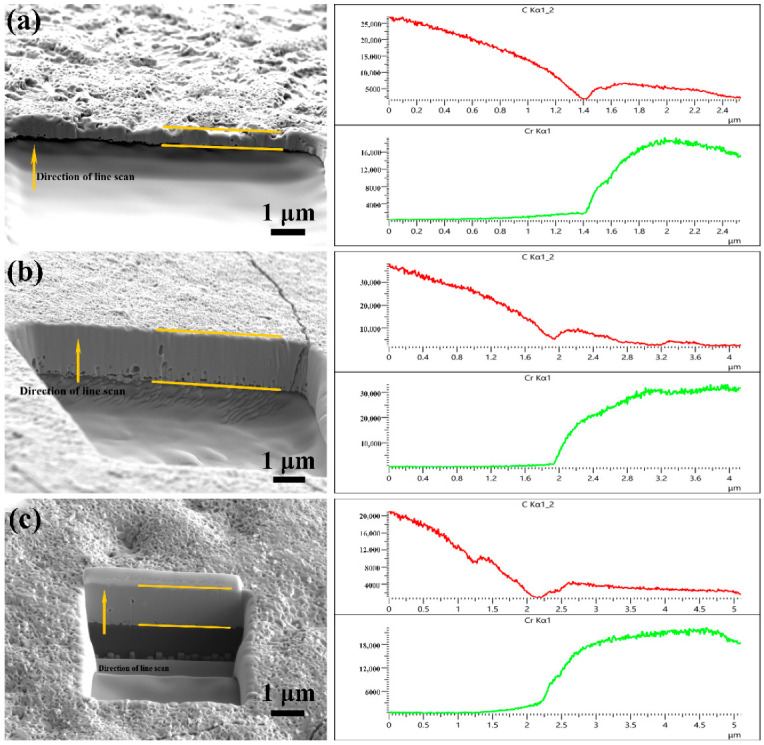

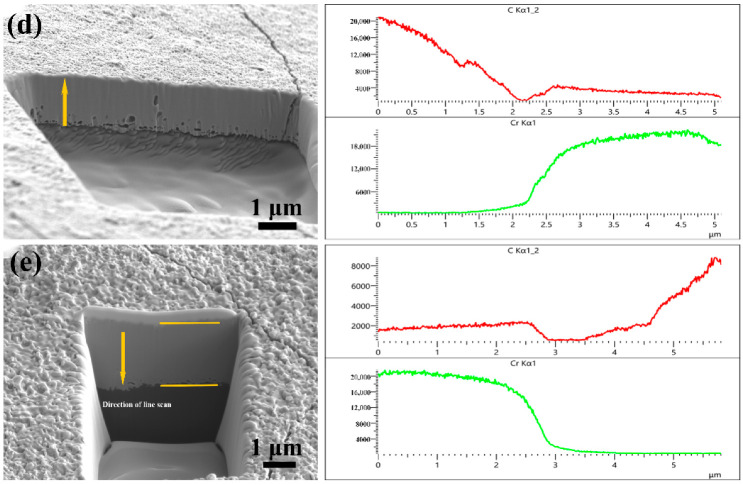
Micromorphology and EDS linear analysis of the coating with various coating parameters: (**a**) 950 °C/30 min, (**b**) 950 °C/60 min, (**c**) 950 °C/90 min, (**d**) 1000 °C/30 min, and (**e**) 1050 °C/30 min.

The SEM image and EDS line scan analysis results of the coating are shown in Figure 3, revealing that the thickness of the coating increased significantly with the increase in temperature but did not change significantly with the holding time. The EDS line scan was performed from the inside to the outside, suggesting that the C content gradually decreased while the Cr content gradually increased. It can be observed that the C content tended to increase slightly after the decrease, which is because some of the C atoms in the diamond and some of the Cr atoms in the coating diffused into each other at the interface, thus reasonably forming carbides on both sides of the interface. The Cr element was observed at 1.09 μm, 1.20 μm, 1.85 μm, 2.50 μm, and 2.95 μm in the coatings at the three temperatures; accordingly, when the coating temperature was 950 °C/30 min, 950 °C/60 min, 950 °C/90 min, 1000 °C/30 min, and 1050 °C/30 min, the coating thickness was 1.09 μm, 1.20 μm, 1.85 μm, 2.50 μm, and 2.95 μm, respectively.

**Figure 4:**
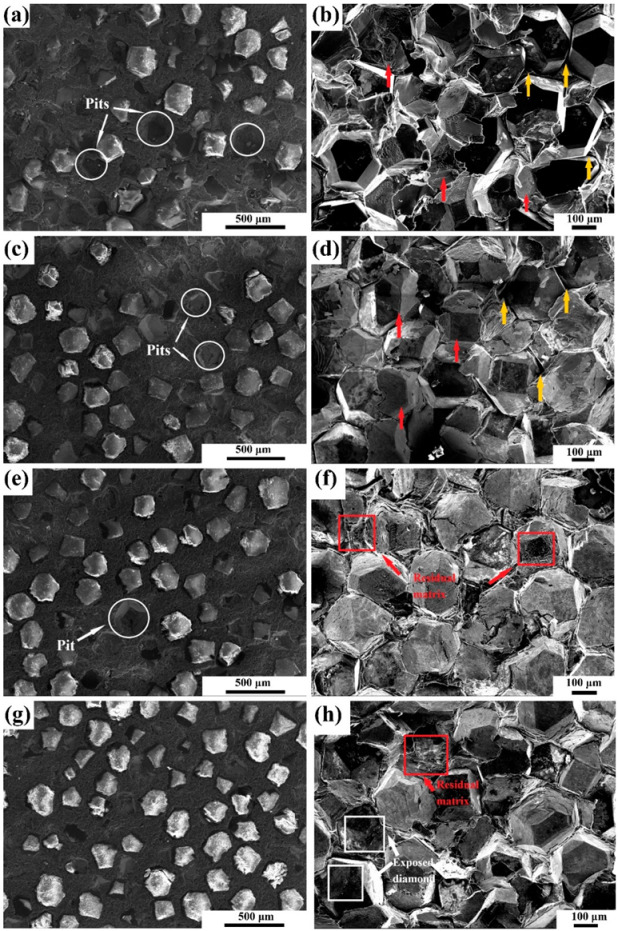

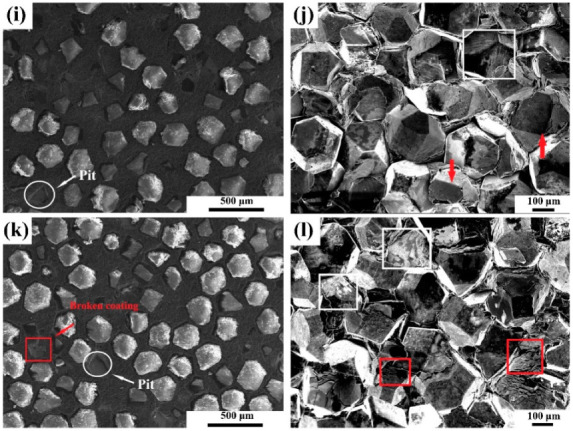
Microstructure and fracture surface of Mg/diamond composite with different coating temperatures: (**a**,**b**) uncoated, (**c**,**d**) 950 °C/30 min, (**e**,**f**) 950 °C/60 min, (**g**,**h**) 950 °C/90 min, (**i**,**j**) 1000 °C/30 min, and (**k**,**l**) 1050 °C/30 min.

The microstructure of the composite material was characterized by SEM. As shown in Figure 4, in the uncoated Mg/diamond composite, the distribution of diamond particles in the matrix was uneven, and there were many pits on the surface. Moreover, the fracture surface shows that there were several holes and voids, and diamond particle shedding resulted in dimples. However, diamond particles were homogeneously distributed and tightly bound in the composites after Cr coating. The introduction of the carbide layer improved the interface bonding, but the mismatch in thermal expansion coefficient between the thick coating and diamond resulted in poor bonding, which indirectly affected the interface bonding strength of the composite and even led to severe interface separation. On the basis of the investigation of the fracture surfaces of composites, it can be speculated that the effect of holding time on the interfacial bond strength of the composites was less significant compared to the influence of coating temperature.

**Figure 5:**
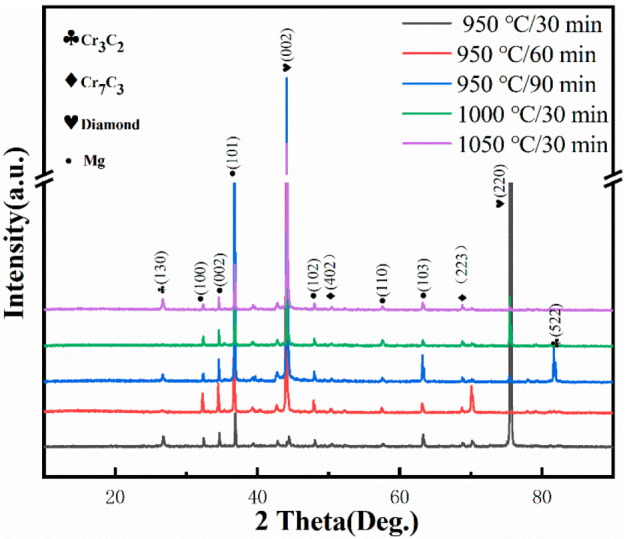
XRD patterns of Mg/diamond (Cr) composites with various coating parameters.

To study the phase composition of Mg/diamond (Cr) composites at different coating temperatures, phase analysis was carried out by XRD, as shown in Figure 5. Cr_3_C_2_, Cr_7_C_3_, diamond, and Mg were identified in all composite samples. Surprisingly, the intensity of Cr_7_C_3_ was significantly lower than that of Cr_3_C_2_, which may be because the residual Cr atoms continued to undergo carbide transformation during the insulation stage of the Mg/diamond composite, thus generating a portion of Cr_3_C_2_. In particular, the Cr_3_C_2_ phase was observed with an intense peak at 82.2° in the XRD pattern when the temperature was kept at 950 °C for 90 min, indicating that, as the holding time increased, the interface layer continued to transform to carbides with high C content.

**Figure 6:**
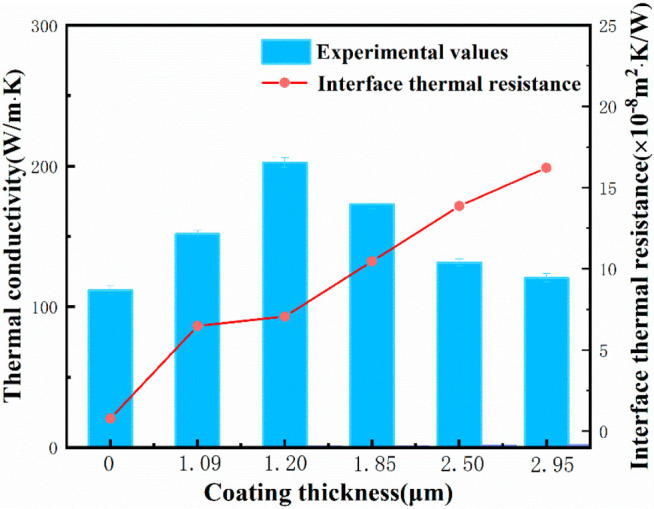
Thermal conductivity and interface thermal resistance of Mg/diamond (Cr) composites with different thicknesses of carbides.

The TC of the Mg/diamond (Cr) composite increased first and then decreased as the thickness of the coating increased, as shown in Figure 6, attaining a maximum TC of 202.42 W/(m·K) at 1.20 μm coating thickness when 950 °C/60 min. The TC of the uncoated Mg/diamond composite was only 111.77 W/ (m·K), owing to the poor wettability between the Mg matrix and the diamond. The coating thickness affects the thermal conductivity Mg/diamond (Cr) composite to a certain extent, whereby a suitable coating thickness can improve the interface bonding, but an excessively thick coating can bring about a larger interface thermal resistance. In other words, the negative effect of the interface thermal resistance is stronger than the positive effect of the carbide layer, resulting in a decrease in thermal conductivity. In addition, according to the results of the interface analysis of the Mg/diamond (Cr) composite, the coating obtained at high temperature already had defects such as cracking and peeling, which would form pores at the Mg/diamond (Cr) composite interface or even result in a discontinuous interface. In the heat transfer process, the interface defects can cause serious scattering effects on the coupling of electrons and phonons, as well as weaken the thermal conductivity of the composite. The thermal conductivity of the Mg/diamond composite with a coating thickness of 2.95 μm was only 120.6 W/ (m·K), which is consistent with the differential effective medium (DEM) model analysis [28].

(1) $$1-V_{p}{\left(\frac{K_{c}}{K_{m}}\right)}^{\frac{1}{3}}=\frac{K_{p}^{eff}-K_{c}}{K_{p}^{eff}-K_{m}}\;\mathrm{with}\;K_{p}^{eff}=\frac{K_{p}}{1+2\frac{K_{p}}{hd}},$$

where $K_{c},\;K_{m},\;\mathrm{and}\;K_{p}$ are the thermal conductivities of the composite, matrix, and reinforcement, respectively. $V_{p}$ and $K_{p}^{eff}$ are the volume fraction of the reinforcement and actual thermal conductivity of the reinforcement, respectively. As calculated using the differential effective medium (DEM) model, the interface thermal resistance of the Mg/diamond (Cr) composite increased as the thickness of the coating increased, and the theoretical thermal conductivity of the Mg/diamond (Cr) composite gradually decreased. Theoretical calculations of the thermal conductivity were much higher than the experimental values, with even the TC of the unmodified Mg/diamond composite reaching 878 W/(m·K). This may be because of Cr_3_C_2_ and Cr_7_C_3_ coexisting in the coating, whereas the DEM model did not take into account the interface defects.

**Figure 7:**
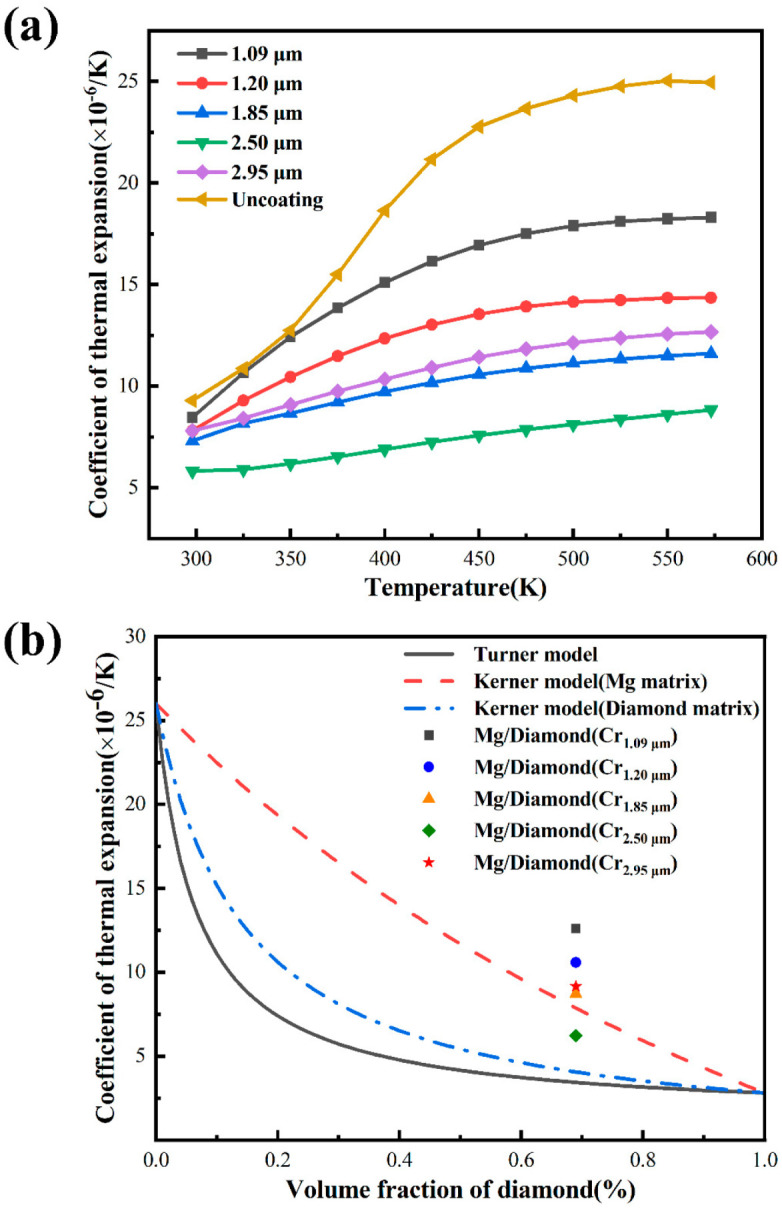
(**a**) Thermal expansion coefficient of Mg/diamond (Cr) composites; (**b**) theoretical calculation and experimental analysis results of thermal expansion coefficient of Mg/diamond (Cr) compo-sites.

The CTE of the Mg/diamond(Cr) and the unmodified Mg/diamond composites was measured, as shown in Figure 7a. The CTE of the uncoated Mg/diamond composite was as high as 24.95 × 10^−6^/K at 573 K. This was attributed to the extreme disparity in the CTE values of diamond (1.3 × 10^−6^/K) [6] and Mg (27.3 × 10^−6^/K) [29], which could easily lead to rapid expansion of the matrix under the condition of poor interface bonding. The CTE of the Mg/diamond (Cr) composite was only 5.82 × 10^−6^/K at 2.50 μm coating thickness, and, with the increase in temperature, the rate of increase in CTE was significantly slower, suggesting that a strong interface bond was formed between the interfaces of the composite at 1000 °C/30 min. The CTE value of the composite material was higher at 2.95 μm coating thickness. This was due to the interface layer carbide itself having brittleness. The excessively thick coating structure caused the interface layer to crack, resulting in a separation of the interface between the matrix and the reinforcement.

Figure 7b shows the measured and theoretically expected CTE values of Mg/diamond composites. The Turner model and Kerner model were applied to calculate the CTE of Mg/diamond composites.

The Turner model [30]:

(2) $$\alpha_{c}=\frac{\alpha_{m}V_{m}K_{m}+\alpha_{p}V_{p}K_{p}}{K_{m}V+K_{p}V_{p}}.$$

The Kerner model [31]:

(3) $$\alpha_{c}=\alpha_{m}V_{m}+\alpha_{p}V_{p}+V_{p}V_{m}\left(\alpha_{p}-\alpha_{m}\right)\times \frac{K_{p}-K_{m}}{V_{m}K_{m}+V_{p}K_{p}+\left(\frac{3K_{p}K_{m}}{4G_{m}}\right)}.$$

The parameters used for calculations were as follows: $K_{m}$ = 35.83 GPa, $K_{p}$ = 580 GPa, $\alpha_{m}$ = 26 × 10^−6^/K, $\alpha_{p}$ = 2.8 × 10^−6^/K, $G_{Mg}$ = 18.2 GPa, and $G_{Diamond}$ = 360 GPa [32]. In Figure 7b, the prediction result of the Turner model under the same conditions was significantly smaller than that of the Kerner model, because the more idealized Turner model ignores the shear stress at which a material undergoes thermal expansion. Furthermore, all the experimental results were higher than the calculated ones, with the only exception being the composites with an interface thickness of 2.50 μm, which fell within the prediction area of the Kerner model. This can be explained by the weak interfacial strength between Mg and the diamond particles. Therefore, an appropriate thickness of the interfacial layer can further improve the interface bonding of the composite, thus broadening the application fields of magnesium matrix composites.

## 4. Conclusions

In this paper, the effect of the Cr coating on the thermal properties of the Mg/diamond composites was investigated. The following conclusions were drawn:

1. The synthesis of the chrome carbide coatings was carried out using a molten salt process. Starting with a coating temperature of 950 °C and a holding time of 30 min, a relatively complete coating was formed on the diamond surface. The coating process at 950 °C/30 min, 950 °C/60 min, 950 °C/90 min, 1000 °C/30 min, and 1050 °C/30 min could obtain an interface layer with a thickness in the range 1.09–2.95 μm.
2. The density and thermal conductivity of the composite reached a maximum value of 97.24% and 202.42 W/(m·K), respectively, compared with the unmodified Mg/diamond composite, thus improving by 6.4% and 81.1%, respectively.
3. The CTE of the Mg/diamond (Cr) composite first decreased and then increased with the increase in coating thickness, reaching a minimum value of 5.82 × 10^−6^/K at 2.50 μm coating thickness, which was only 60% of the CTE of the uncoated Mg/diamond composite, thus effectively matching the thermal expansion coefficient of the semiconductor material.

In the molten salt process, due to the difference in surface energy between the diamond (100) and (111) surfaces, the deposition rate of carbides on the (100) is faster than on the (111) surface. If the coating temperature is too high and the holding time is too long, the coating on the (100) crystal surface will crack because it does not match the CTE of diamond.

The Mg/diamond (Cr) composite was prepared using the squeeze casting infiltration process under a pressure of 10 MPa. The density and thermal conductivity of the composite first increased and then decreased with the increase in coating thickness, mainly because the conversion of carbides could optimize the interface bonding, and the presence of thicker coatings and interface defects would produce higher interface thermal resistance, resulting in a decrease in thermal conductivity.

## Figures and Tables

**Table 1:** Raw materials and experimental parameters.

Raw Materials				Experimental Parameters
	purity	Size (μm)	TC value (W/(m·K))	Coating time–30–90 min
				Coating temperature—950–1050 °C
Diamond	-	230	1800	Preparation temperature—800 °C
Mg	>99.95%	-	156	holding time—10 min
Cr	>99.99%	70	-	infiltration pressure—10 MPa
NaCl	>99.99%	-	-	infiltration time—60 s
KCl	>99.99%	-	-	graphite mold—φ20 mm × 4 mm

**Table 2:** Gibbs energies of formation of chromium carbides.

Chemical Equations	Gibbs Functions
$3Cr_{\left(s\right)}+2C_{\left(s\right)}=Cr_{3}C_{2}{}_{\left(s\right)}$	$∆G_{T}^{0}=-95.95-0.0198T$(KJ/mol)
$7Cr_{\left(s\right)}+3C_{\left(s\right)}=Cr_{7}C_{3}{}_{\left(s\right)}$	$∆G_{T}^{0}=-182.5-0.0429T$(KJ/mol)
$23Cr_{\left(s\right)}+6C_{\left(s\right)}=Cr_{23}C_{6}{}_{\left(s\right)}$	$∆G_{T}^{0}=-398.43-0.0827T$(KJ/mol)

## Data Availability

Data are contained within the article.

## Nomenclature

Symbols and acronyms	
TC	thermal conductivity
CET	coefficient of thermal expansion
MMCs	metal matrix composites
$K_{c}$	thermal conductivity of the Mg/diamond (Cr) composite (W/(m·K))
α	thermal diffusivity (m^2^/s)
$\rho_{c}$	density of the Mg/diamond (Cr) composite (g/cm^3^)
c	heat capacity (J·g^−1^·K^−1^)

## References

[1] Razeeb K.M., Dalton E., Cross G.L.W., Robinson A.J. (2018). Present and future thermal interface materials for electronic devices. Int. Mater. Rev..

[2] Zhang S., Xu X., Lin T., He P. (2019). Recent advances in nano-materials for packaging of electronic devices. J. Mater. Sci. Mater. Electron..

[3] Dadkhah M., Saboori A., Fino P. (2019). An overview of the recent developments in metal matrix nanocomposites reinforced by graphene. Materials.

[4] Shirvanimoghaddam K., Hamim S.U., Akbari M.K., Fakhrhoseini S.M., Khayyam H., Pakseresht A.H., Ghasali E., Zabet M., Munir K.S., Jia S. (2017). Carbon fiber reinforced metal matrix composites: Fabrication processes and properties. Compos. Part A Appl. Sci. Manuf..

[5] Inyushkin A., Taldenkov A., Ralchenko V., Bolshakov A., Koliadin A., Katrusha A. (2018). Thermal conductivity of high purity synthetic single crystal diamonds. Phys. Rev. B.

[6] Jacobson P., Stoupin S. (2019). Thermal expansion coefficient of diamond in a wide temperature range. Diam. Relat. Mater..

[7] Molina-Jorda J.M. (2015). Nano- and micro-/meso-scale engineered magnesium/diamond composites: Novel materials for emerging challenges in thermal management. Acta Mater..

[8] Molina-Jorda J.M. (2018). Multi-scale design of novel materials for emerging challenges in active thermal management: Open-pore magnesium-diamond composite foams with nano-engineered interfaces. Compos. Part A Appl. Sci. Manuf..

[9] Pan Y., He X., Ren S., Wu M., Qu X. (2018). Optimized thermal conductivity of diamond/Cu composite prepared with tungsten-copper-coated diamond particles by vacuum sintering technique. Vacuum.

[10] Wang L., Li J., Catalano M., Bai G., Li N., Dai J., Wang X., Zhang H., Wang J., Kim M.J. (2018). Enhanced thermal conductivity in Cu/diamond composites by tailoring the thickness of interfacial TiC layer. Compos. Part A Appl. Sci. Manuf..

[11] Zhu C.X., Cui C., Wu X.W., Zhang B.W., Yang D., Zhao H.X., Zheng Z. (2020). Study on surface modification of diamond particles and thermal conductivity properties of their reinforced metal-based (Cu or Mg) composites. Diam. Relat. Mater..

[12] Xie Z., Guo H., Zhang X., Huang S., Xie H., Mi X. (2021). Tailoring the thermal and mechanical properties of diamond/Cu composites by interface regulation of Cr alloying. Diam. Relat. Mater..

[13] Yang L., Sun L., Bai W., Li L. (2019). Thermal conductivity of Cu-Ti/diamond composites via spark plasma sintering. Diam. Relat. Mater..

[14] Li J., Wang X., Qiao Y., Zhang Y., He Z., Zhang H. (2015). High thermal conductivity through interfacial layer optimization in diamond particles dispersed Zr-alloyed Cu matrix composites. Scr. Mater..

[15] Kumar C.M.P., Chandrashekarappa M.P.G., Kulkarni R.M., Pimenov D.Y., Giasin K. (2021). The Effect of Zn and Zn-WO3 Composites Nano-Coatings Deposition on Hardness and Corrosion Resistance in Steel Substrate. Materials.

[16] Kumar C.M.P., Lakshmikanthan A., Chandrashekarappa M.P.G., Pimenov D.Y., Giasin K. (2021). Electrodeposition Based Preparation of Zn-Ni Alloy and Zn-Ni-WC Nano-Composite Coatings for Corrosion-Resistant Applications. Coatings.

[17] Ying T., Chi H., Zheng M., Li Z., Uher C. (2014). Low-temperature electrical resistivity and thermal conductivity of binary magnesium alloys. Acta Mater..

[18] Polat S. (2022). Theoretical modeling and optimization of interface design to improve thermal conductivity in Mg-Dia composites. Ceram. Int..

[19] Náprstková N., Novák M., Marek M., Šramhauser K., Sviantek J., Stančeková D., Ťavodová M. (2021). Analyses of Influence on Chromium Coating after Grinding from the View of Final Microstructure and Microhardness in the Surface Layer. Materials.

[20] Ren S., Chen J., He X., Qu X. (2018). Effect of matrix-alloying-element chromium on the microstructure and properties of graphite flakes/copper composites fabricated by hot pressing sintering. Carbon.

[21] Stevenson R.D., Whatley W.J., Glatz J.J., McCoy J.W. (1993). Aerospace, Defense, and Demanding Applications. Proceedings of the 3rd International Conference on Powder Met.

[22] Pickard S.M., Withers J.C., Loufty R.O. (2014). High Thermal Conductivity Metal Matrix Composites. E.P. Patent.

[23] Ma H., Wang J., Wang H., Dong N., Zhang J., Jin P., Peng Y. (2021). Influence of nano-diamond content on the microstructure, mechanical and thermal properties of the ZK60 composites. J. Magnes. Alloys.

[24] Behboudi F., Kakroudi M.G., Vafa N.P., Faraji M., Milani S.S. (2021). Molten salt synthesis of in-situ TiC coating on graphite flakes. Ceram. Int..

[25] Jia J.H., Bai S.X., Xiong D.G., Wang J., Chang J. (2019). Effect of tungsten based coating characteristics on microstructure and thermal conductivity of diamond/Cu composites prepared by pressueless infiltration. Ceram. Int..

[26] Chu K., Jia C., Guo H., Li W. (2012). Microstructure and thermal conductivity of Cu-B/diamond composites. J. Compos. Mater..

[27] Tan Z.Q., Li Z.Q., Fan G.L., Guo Q., Kai X.Z., Ji G., Zhang L.T., Zhang D. (2013). Enhanced thermal conductivity in diamond/aluminum composites with a tungsten interface nanolayer. Mater. Des..

[28] Tavangar R., Molina J.M., Weber L. (2007). Assessing predictive schemes for thermal conductivity against diamond-reinforced silver matrix composites at intermediate phase contrast. Scr. Mater..

[29] Martienssen W., Warlimont H. (2006). Springer Handbook of Condensed Matter and Materials Data.

[30] Turner P.S. (1942). The Problem of Thermal-Expansion Stresses in Reinforced Plastics. https://ntrs.nasa.gov/citations/19930093345.

[31] Kerner E. (1956). The elastic and thermo-elastic properties of composite media. Proc. Phys. Soc. Sect. B.

[32] Yoshida K., Morigami H. (2004). Thermal properties of diamond/copper composite material. Microelectron. Reliab..

